# Effects of the cucumber mosaic virus 2a protein on aphid–plant interactions in *Arabidopsis thaliana*

**DOI:** 10.1111/mpp.12975

**Published:** 2020-07-28

**Authors:** Sun‐Ju Rhee, Lewis G. Watt, Ana Cazar Bravo, Alex M. Murphy, John P. Carr

**Affiliations:** ^1^ Department of Plant Sciences University of Cambridge Cambridge UK

**Keywords:** antixenosis, CMV strain difference, host manipulation, nonpersistent, vector, viral replicase protein, virus transmission

## Abstract

The cucumber mosaic virus (CMV) 2a RNA‐dependent RNA polymerase protein has an additional function in *Arabidopsis thaliana*, which is to stimulate feeding deterrence (antixenosis) against aphids. Antixenosis is thought to increase the probability that aphids, after acquiring CMV particles from brief probes of an infected plant's epidermal cells, will be discouraged from settling and instead will spread inoculum to neighbouring plants. The amino acid sequences of 2a proteins encoded by a CMV strain that induces antixenosis in *A. thaliana* (Fny‐CMV) and one that does not (LS‐CMV) were compared to identify residues that might determine the triggering of antixenosis. These data were used to design reassortant viruses comprising Fny‐CMV RNAs 1 and 3, and recombinant CMV RNA 2 molecules encoding chimeric 2a proteins containing sequences derived from LS‐CMV and Fny‐CMV. Antixenosis induction was detected by measuring the mean relative growth rate and fecundity of aphids (*Myzus persicae*) confined on infected and on mock‐inoculated plants. An amino acid sequence determining antixenosis induction by CMV was found to reside between 2a protein residues 200 and 300. Subsequent mutant analysis delineated this to residue 237. We conjecture that the Fny‐CMV 2a protein valine‐237 plays some role in 2a protein‐induced antixenosis.

## Graphical abstract

Residue 237 of the cucumber mosaic virus 2a protein plays a role in induction of feeding deterrence (antixenosis), which encourages viruliferous aphids to disperse to uninfected hosts.

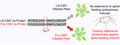

Cucumber mosaic virus (CMV) is an insect‐transmitted virus that modifies interactions between its infected host plants and its aphid vectors in ways that increase the probability of transmission over various ranges and timescales (Donnelly *et al*., 2019; Carr *et al*., 2020). CMV does not infect its aphid vectors but influences their behaviour by altering the biochemistry of infected host plants. The paradigmatic example of this phenomenon is the interaction of the aphids *Aphis gossypii* and *Myzus persicae* with *Cucurbita pepo* plants infected with the Fny strain of CMV (Fny‐CMV) (Mauck *et al*., 2010). CMV infection causes infected cucurbits to emit a mix of volatile organic compounds that attract aphids, but infection also induces accumulation of antixenotic, that is, feeding‐deterrent, compounds in the leaves that ensure that aphids feed for only a brief time before moving to another plant (Mauck *et al*., 2010; Carmo‐Souza *et al*., 2014). Because CMV is a nonpersistently transmitted virus (virus particles acquired from an infected plant are attached loosely to an aphid's stylet mouthparts), this short feed is sufficient to render the aphids competent to transmit infection to neighbouring hosts (Krenz *et al*., 2015). A similar phenomenon has been observed in the interactions between *M. persicae* and plants of the model species *Arabidopsis thaliana* infected with Fny‐CMV (Westwood *et al*., 2013). Using this system, it was found that antixenosis was induced in some fashion by the CMV 2a protein (Westwood *et al*., 2013). Plants possess a variety of mechanisms that enable them to resist aphid infestation or discourage feeding by these insects (Nalam *et al*., 2019). In the case of CMV‐infected *A. thaliana*, Westwood *et al*. (2013) proposed that the 2a protein induces production of the antixenotic compound 4‐methoxy‐indol‐3‐yl‐methylglucosinolate (Kim and Jander, 2007; Mewis *et al*., 2012) by activation of the pathogen‐associated molecular pattern‐triggered immunity system.

The primary function of the CMV 2a protein is to act as the viral RNA‐dependent RNA polymerase, which catalyses synthesis of new genomic and subgenomic RNA molecules (Palukaitis and García‐Arenal, 2003; Seo *et al*., 2019). The 2a protein is one of five proteins encoded by the tripartite, positive‐sense RNA genome of CMV. RNA 1 is translated directly to yield the 1a methyltransferase/helicase protein, which associates with the 2a protein during replicase complex formation (Palukaitis and García‐Arenal, 2003; Seo *et al*., 2019). The 97 kDa 2a protein is translated from the 5′‐proximal open reading frame (ORF) of RNA 2. An overlapping ORF encodes the 2b counterdefence protein (Figure 1a), which is expressed from a viral subgenomic mRNA (RNA 4A) (Palukaitis and García‐Arenal, 2003). CMV RNA 3 acts as a translation template for the viral moment protein and also encodes the viral coat protein, which is expressed by translation of subgenomic RNA 4 (Palukaitis and García‐Arenal, 2003). The coat protein is the sole viral factor needed for attachment of CMV particles to acrostyle receptors in the aphid stylet (Webster *et al*., 2018; Fereres and Perry, 2019).

**Figure 1:**
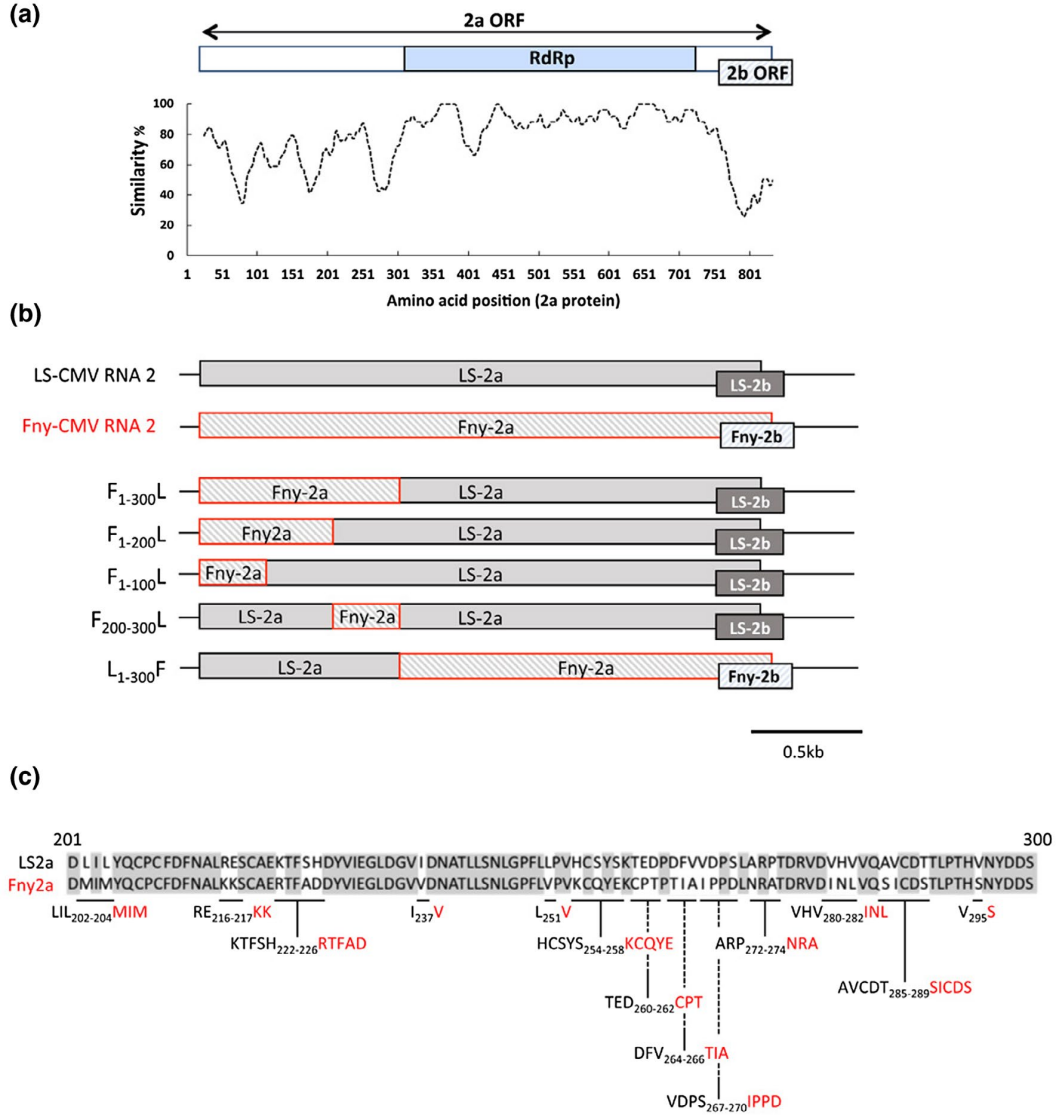
Design of recombinant CMV RNA 2 molecules to identify CMV 2a protein sequences that influence plant–aphid interactions. (a) Amino acid sequence alignment and similarity between the Fny‐CMV and LS‐CMV 2a proteins. The line graph illustrates the degree of similarity obtained using plotcon (EMBOSS Explorer: http://emboss.bioinformatics.nl/cgi‐bin/emboss/plotcon) with a window size of 10 residues. A diagram of the 2a protein ORF is displayed above the similarity plot showing the RNA‐dependent RNA polymerase (RdRp) domain and the overlapping 2b ORF. (b) Five recombinant CMV RNA 2 molecules were produced by ligating together segments of the LS‐CMV and Fny‐CMV RNA 2 molecules to produce in‐frame 2a ORFs encoding chimeric 2a proteins (Methods S1). Sequences derived from the Fny‐CMV 2a protein ORF are depicted with a red outline, and construct names are based on the sequence coordinates (in subscript text) for residues derived from each strain's 2a protein, for example the 2a ORF of F_200‐300_L encodes the Fny‐CMV 2a amino acid sequence between residues 200 and 300, with the remainder derived from the LS‐CMV 2a protein sequence. (c) The amino acid sequences for the LS‐CMV and Fny‐CMV 2a proteins between residues 201 and 300 are shown. Shading indicates amino acid sequence conservation between the two 2a protein sequences. Site‐directed mutagenesis was used to substitute LS‐CMV RNA 2 sequences with those from Fny‐CMV RNA 2 at 13 sites and the resulting changes in amino acid sequence are indicated in red

In a previous study of the effects of the CMV 2a protein on interactions of *A. thaliana* Col‐0 and *M. persicae* (isolate USL1: Devonshire and Sawicki, 1979), the mean relative growth rate (MRGR) of aphid nymphs confined on plants had been used as the sole proxy for aphid performance (Westwood *et al*., 2013). However, as shown with tobacco, virus‐induced changes in MRGR do not always correlate with decreased reproduction (Ziebell *et al*., 2011). Nevertheless, on *2a*‐transgenic *A. thaliana* plants the MRGR and colony growth of *M. persicae* (mean number of offspring produced per aphid) were both impeded, showing that the Fny‐CMV 2a protein induces effects in this plant that decrease both measures of performance (Figure S1 and Spreadsheet S1). Therefore, in this study we used both assays to control for the possibility that different sequences within the CMV 2a protein influence different aspects of CMV‐induced antixenosis.

In this study we sought to identify amino acid residue(s) in the CMV 2a protein involved in induction of antixenosis in CMV‐infected *A. thaliana* plants. Our approach took advantage of our finding that although the Fny strain of CMV induces antixenosis in *A. thaliana*, the strain LS‐CMV does not (Westwood *et al*., 2013). Comparison of the amino acid sequences of the 2a proteins encoded by each strain enabled us to begin delineating which residue or residues might determine antixenosis induction (Figure 1a). The amino acid sequences of the RNA‐dependent RNA polymerase domains showed the highest degrees of conservation (Figure 1a). The greatest dissimilarity between the two 2a proteins occurs in the N‐proximal 300 residues, and in the C‐terminal regions of the 2a proteins, which correspond to the region of the 2a ORF that overlaps with part of the ORF encoding the 2b protein (Figure 1a). Although the region of the 2a ORF that overlaps with the 2b ORF has a number of effects on the pathology and movement of CMV (Du *et al*., 2008; Khaing *et al*., 2020), it is not required for antixenosis induction in *A. thaliana* (Westwood *et al*., 2013). Therefore, we hypothesized that the region spanning residues 1–300 of the 2a protein is the most likely to contain amino acid(s) that determine antixenosis induction. Five recombinant cDNA clones encoding chimeric RNA 2 molecules were constructed in which the regions encoding all or part of the N‐proximal 300 residues of the 2a protein comprised sequences exchanged between the RNA 2 sequences of Fny‐CMV and LS‐CMV (Figure 1b). Constructs were derived from plasmids pFny206 and pLS‐CMV2, the respective infectious cDNA clones for the Fny‐CMV and LS‐CMV RNA 2 molecules (Rizzo and Palukaitis, 1990; Zhang *et al*., 1994) (Table S1). Wild‐type or recombinant RNA 2 molecules were synthesized by in vitro transcription using T7 RNA polymerase, and infectious RNA mixtures produced by mixing these with in vitro‐synthesized Fny‐CMV RNAs 1 and 3. Infectious RNA mixtures for these reassortant and recombinant viruses were used to inoculate *Nicotiana benthamiana* plants for preparation of virions to use as inocula for experiments with *A. thaliana* (Palukaitis, 2019). We recently used a similar approach with Fny‐CMV/LS‐CMV reassortants to successfully identify the viral RNA conditioning antibiosis (strong resistance) against aphids in *Nicotiana tabacum* (Tungadi *et al*., 2020). RNA was isolated from systemically infected leaves, subjected to reverse transcription‐PCR (RT‐PCR) to amplify RNA 2‐specific sequences and these amplicons sequenced to confirm that all recombinant RNA 2 molecules were genetically stable and did not undergo further mutation during replication and movement through the plant. Plants of *A. thaliana* and *N. benthamiana* infected with viruses containing recombinant RNA 2 molecules exhibited easily discernable systemic disease symptoms that were less severe than those induced by reconstituted Fny‐CMV (F1‐F2‐F3: generated by mixing Fny‐CMV RNAs 1, 2, and 3), and similar in severity to symptoms induced by the reassortant virus F1‐L2‐F3 (constituted from Fny‐CMVs RNAs 1 and 3, and LS‐CMV RNA 2) (Figure S2 and Spreadsheet S2), and accumulated to similar levels in *A. thaliana* (Figure S3a).

*A. thaliana* plants were used for aphid performance experiments at 10 days following either inoculation with virions or mock inoculation with water. The induction of resistance to *M. persicae* confined on plants was assessed using two measures of aphid performance: MRGR of aphid nymphs over the first 6 days of life and subsequent reproduction. Both assays were carried out using the same batches of aphids and plants, and carried out on three separate occasions (Figure 2). MRGR was decreased for aphid nymphs placed on plants infected with F1‐F2‐F3, but not with the reassortant virus F1‐L2‐F3 (Figure 2). This is consistent with the conclusion of Westwood *et al*. (2013) that the 2a protein induces feeding deterrence, resulting in aphid growth inhibition. In this study it was found that aphid reproduction was also inhibited on plants infected with F1‐F2‐F3, but not on plants infected with the F1‐L2‐F3 reassortant virus (Figure 2).

**Figure 2:**
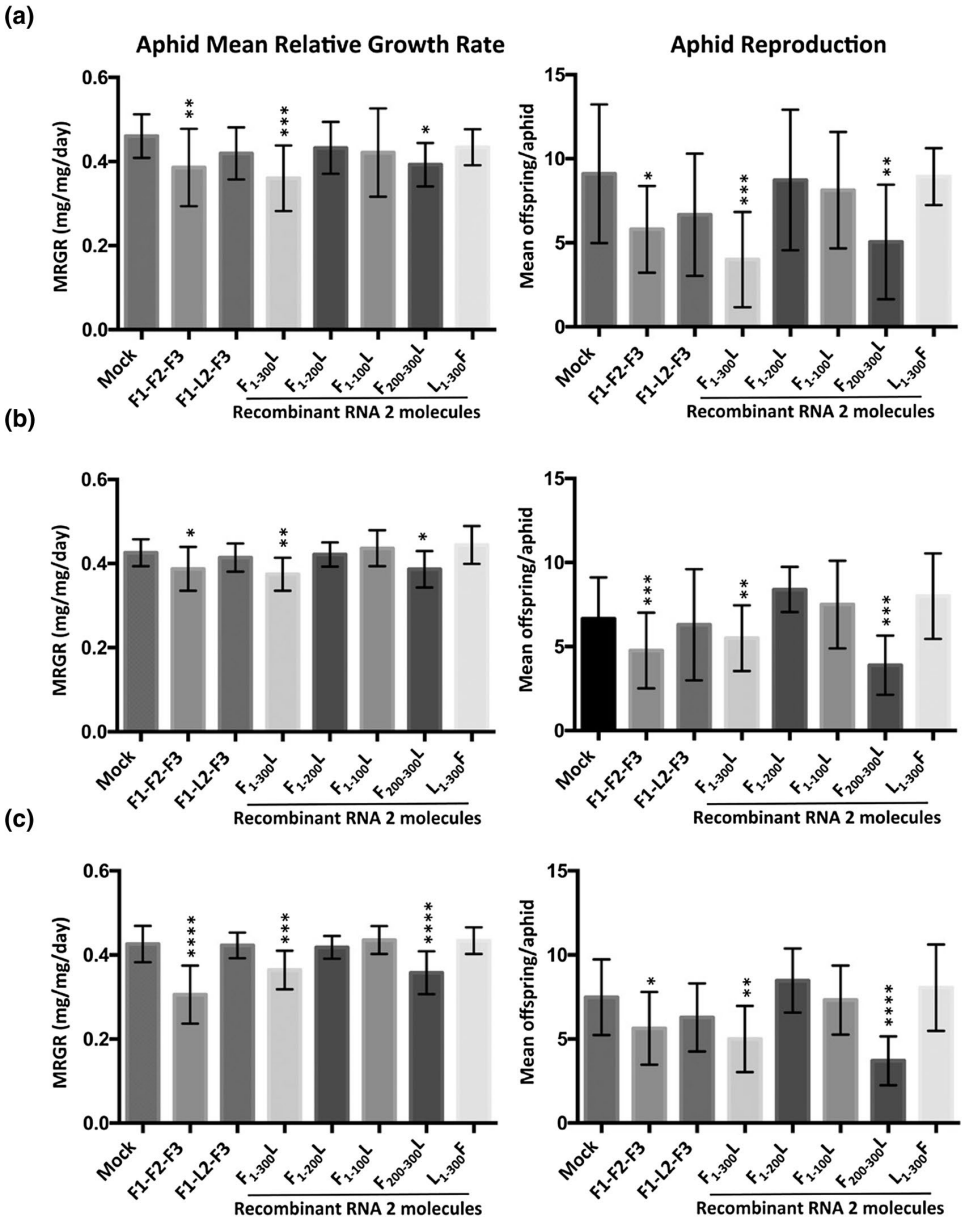
Performance of *Myzus persicae* on *Arabidopsis thaliana* plants infected with Fny‐CMV/LS‐CMV reassortant viruses with Fny‐CMV/LS‐CMV recombinant RNA 2 molecules. One‐day‐old aphid nymphs (10–12 aphids per treatment per experiment) were weighed using a microbalance, placed on plants, reweighed 5 days later, and the mean relative growth rate (MRGR) calculated as previously described (Leather and Dixon, 1984; Stewart *et al*., 2009; Ziebell *et al*., 2011; Westwood *et al*., 2013) (histograms on left). Ten days later the offspring produced by each aphid were counted and the mean colony size calculated (Ziebell *et al*., 2011; Westwood *et al*., 2013; Tungadi *et al*., 2020) (histograms on right). Plants were mock‐inoculated (Mock) with sterile water or inoculated with purified virions of reconstituted Fny‐CMV (F1‐F2‐F3), the reassortant Fny‐CMV/LS‐CMV virus F1‐L2‐F3, or viruses possessing RNAs F1, F3, and one of the indicated Fny‐CMV(F)/LS‐CMV(L) recombinant RNA 2 molecules (described in Figure 1b). Plants were used for aphid performance experiments at 10 days postinoculation. Panels (a), (b), and (c) are the results of three independent experiments. Error bars represent standard error around the mean. Statistical analysis was performed using one‐way analysis of variance followed by Dunnett's post hoc multiple comparisons test (significant differences indicated by ****p < .0001; ***p < .001; **p < .01, and *p < .01) using R (Dalgaard, 2008)

The RNA 2 of F1‐L_1‐300_F‐F3 possesses a 2a ORF in which residues 1–300 are derived from LS‐CMV RNA 2 (Figure 1b). Neither aphid growth nor fecundity was affected on plants infected with the reassortant/recombinant virus F1‐L_1‐300_F‐F3 (Figure 2). The recombinant RNA 2 used to constitute this reassortant virus possesses the ORF for the Fny‐CMV 2b protein (Figure 1b). The Fny‐CMV 2b protein can induce a variety of effects on performance of aphids on tobacco plants infected with Fny‐CMV (Ziebell *et al*., 2011; Tungadi *et al*., 2020) and in *2b*‐transgenic *A. thaliana* plants (Westwood *et al*., 2013). However, because infection with F1‐L_1‐300_F‐F3 did not induce resistance to aphids, it appears that the 2b protein is not conditioning aphid resistance induced in *A. thaliana* by CMV infection. This is consistent with the conclusion of Westwood *et al*. (2013) that the 2a protein conditions feeding deterrence during CMV infection. That F1‐L_1‐300_F‐F3 does not induce resistance to aphids is consistent with our starting hypothesis that the sequence determining aphid resistance induction lies within the region that shows the most dissimilarity between the 2a proteins of Fny‐CMV and LS‐CMV (Figure 1a). Neither growth nor reproduction of aphids was affected on *A. thaliana* plants infected with the reassortant/recombinant viruses F1‐F_1‐200_L‐F3 or F1‐F_1‐100_L‐F3. Conversely, aphid growth and reproduction were decreased on plants infected with F1‐F_1‐300_L‐F3 and F1‐F_200‐300_L‐F3 (Figure 2). These results were not only consistent with the hypothesis that the N‐proximal 300 residues of the Fny‐CMV 2a protein determine aphid resistance induction in *A. thaliana*, but also suggested that residue(s) important in CMV‐induced resistance to aphids lie between positions 200 and 300 in the Fny‐CMV 2a protein sequence.

Comparison of the 2a protein sequences encoded by LS‐CMV and Fny‐CMV revealed 27 differences in the sequence of amino acids lying between residues 200 and 300 (Figure 1c). Using site‐directed mutagenesis, 13 recombinant versions of LS‐CMV RNA 2 were generated in which between one and five codons from the Fny‐CMV 2a ORF sequence were substituted for corresponding codons of the LS‐CMV 2a ORF (Tables 1 and S2, and Figure 1c). RNA for each RNA 2 recombinant was synthesized by in vitro transcription and infectious RNA mixtures constituted by combining with in vitro‐synthesized RNAs 1 and 3 of Fny‐CMV. These mixtures were used to infect *N. benthamiana* plants and virions were purified from systemically infected leaves for use as inocula for experiments with *A. thaliana*. Viral RNA from systemically infected leaves was amplified by RT‐PCR with RNA 2‐specific primers and amplicons sequenced to confirm that the introduced mutations were stable in planta.

**Table 1:** Specific alterations in the primary amino acid sequence of the 2a protein produced by site‐specific mutagenesis of LS‐CMV RNA 2

Amino acid sequence replacement	Specific 2a protein residue(s) replaced
LIL_202‐204_MIM	202 and 204
RE_216‐217_KK	216 and 217
KTFSH_222‐226_RTFAD	222, 225, and 226
I_237_V	237
L_251_V	251
HCSYS_254‐258_KCQYE	254, 256, and 258
TED_260‐262_CPT	260, 261, and 262
DFV_264‐266_TIA	264, 265, and 266
VDPS_267‐270_IPPD	267, 268, and 270
ARP_272‐274_NRA	272 and 274
VHV_280‐282_INL	280, 281, and 282
AVCDT_285‐289_SICDS	285, 286, and 289
V_295_S	295

Note

The amino acid sequence of the 2a protein of LS‐CMV was modified at 13 sites by replacement with corresponding sequences from the Fny‐CMV 2a protein (Table S2). The positions of these modifications within the 2a protein primary sequence are shown graphically in Figure 1c.

Symptoms induced by several of these mutant viruses differed from those induced by the F1‐L2‐F3 reassortant virus, suggesting that this region of the CMV 2a protein or its corresponding RNA sequence may influence symptomology, especially with respect to the effects of infection on leaf shape (Figure S4). There were no statistically significant differences in accumulation between the viruses in *A. thaliana* (Figure S3b). Aphid nymphs were placed on systemically infected plants at 10 days postinoculation, and aphid growth and reproduction were measured as already described, and the effects of each recombinant RNA 2 on aphid performance observed in three to five independent experiments (Spreadsheet S3). Only the I_237_V mutant induced statistically significant decreases in aphid performance (MRGR and colony growth) consistently in three out of three independent experiments (Figure 3 and Spreadsheet S2). The mutant VHV_280‐282_INL induced a statistically significant decrease in aphid performance in one out of three experiments, so two additional experiments were performed with this mutant to confirm that this was not a biologically relevant result (Spreadsheet S3). No other mutations induced statistically significant decreases in aphid performance.

**Figure 3:**
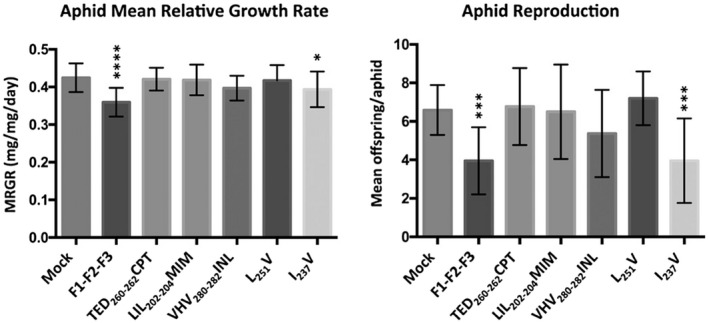
Effects on performance of *Myzus persicae* on *Arabidopsis thaliana* plants of infection with Fny‐CMV/LS‐CMV reassortant viruses with site‐specific mutations in the 2a open reading frame (ORF). The results shown here are for one experiment using a subset of viral mutants. The full range of data, including this experiment (Experiments 2 and 3), for all mutants is available in Spreadsheet S3. One‐day‐old aphid nymphs (10–12 aphids per treatment per experiment) were weighed using a microbalance, placed on plants, reweighed 5 days later, and the mean relative growth rate (MRGR) calculated as previously described (Leather and Dixon, 1984; Stewart *et al*., 2009; Ziebell *et al*., 2011; Westwood *et al*., 2013) (histograms on left). Ten days later the offspring produced by each aphid were counted and the mean number of aphids in each colony calculated (Ziebell *et al*., 2011; Westwood *et al*., 2013; Tungadi *et al*., 2020) (histograms on right). Plants were mock‐inoculated (Mock) with sterile water or inoculated with purified virions of reconstituted Fny‐CMV (F1‐F2‐F3), or versions of the reassortant Fny‐CMV/LS‐CMV virus F1‐L2‐F3, with site‐specific mutations in RNA 2 affecting the 2a ORF (described in Table 1 and Figure 1c). Plants were used for aphid performance experiments at 10 days postinoculation. Error bars represent standard error around the mean. Statistical analysis was performed using one‐way analysis of variance followed by Dunnett's post hoc multiple comparisons test (significant differences indicated by ****p < .0001; ***p < .001; **p < .01, and *p < .01) using R (Dalgaard, 2008)

Thus, we think it probable that the valine at position 237 in the Fny‐CMV 2a protein sequence plays some role in induction of antixenosis against aphids in CMV‐infected *A. thaliana*. That replacement of isoleucine at this position in the LS‐CMV 2a protein sequence with valine had such a marked effect was initially surprising because both amino acids have hydrophobic side chains, making the I_237_V replacement conservative relative to some of the other sequence replacements. However, the literature provides several examples where replacement of isoleucine with valine, or vice versa, has profound effects on the biological activity of proteins. These examples include, among others, the effect of the brome mosaic virus movement protein on symptomology in *N. benthamiana* (Rao and Grantham, 1995), effects on herbicide resistance of the photosynthetic D1 protein (Mengistu *et al*., 2000; Dumont *et al*., 2016), *Escherichia coli rec*A protein function (Knight *et al*., 1984), human 5‐hydroxytryptamine receptor and glutathione S‐transferase activity (Zimniak *et al*., 1994; Nakhai *et al*., 1995), and on β‐amyloid induced neuropathology (Yoshioka *et al*., 1991). It remains unknown how the Fny‐CMV 2a protein valine‐237 might be involved in inducing antixenosis against aphids in *A. thaliana* plants, and we cannot exclude additional roles for other residues of the 2a protein. Our working hypothesis is that this residue directly or indirectly aids an interaction between the Fny‐CMV 2a protein and a host factor or factors involved in either defensive signalling or the regulation of metabolism, leading to increased production of 4‐methoxy‐indol‐3‐yl‐methylglucosinolate and/or other antixenotic compounds.

## CONFLICT OF INTEREST

The authors declare that they have no conflicts of interest.

## Supporting information


**FIGURE S1**

**FIGURE S2**

**FIGURE S3**

**FIGURE S4**

**TABLE S1**

**TABLE S2**

**METHOD S1**

**SPREADSHEET S1**

**SPREADSHEET S2**

**SPREADSHEET S3**

## Data Availability

All relevant data are within the paper and its Supporting Information files.
