# Supplementary material for: Effects of the cucumber mosaic virus 2a protein on aphid–plant interactions in Arabidopsis thaliana
Source: Mol Plant Pathol. 2020 Jul 28;21(9):1248–54. doi: 10.1111/mpp.12975 (PMC7411660; doi:10.1111/mpp.12975)
Supplement: Supplementary file 1 — FIGURE S1 [file MPP-21-1248-s001.pdf]

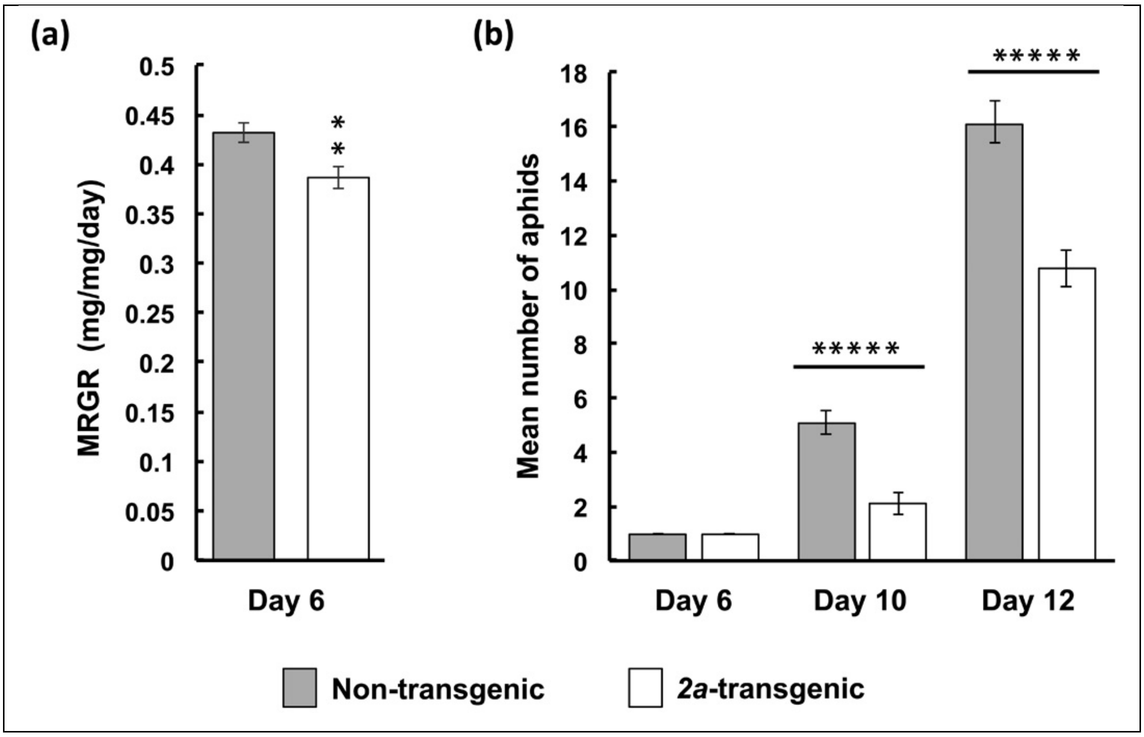

Fig. S1 Aphids confined on transgenic plants expressing the cucumber mosaic virus (CMV) 2a protein exhibit decreases in growth rate and fecundity. A single aphid (*Myzus persicae*) nymph was placed on individual four-week old non-transgenic (NT) *Arabidopsis thaliana* Col-0 plants or on transgenic plants constitutively expressing the CMV 2a protein. The mean relative growth rate (MRGR) was calculated after 6 days after placement (a). The number of progeny produced by each aphid was recorded at 6, 10, and 12 days after placement (b). Error bars represent standard error of the mean (n=16 per treatment) and significant differences between treatments on each day were identified using Student's t-test. Statistically significant differences are indicated by: **, $P < 0.01$, and *****, $P < 0.00001$.
