# Supplementary material for: Effects of the cucumber mosaic virus 2a protein on aphid–plant interactions in Arabidopsis thaliana
Source: Mol Plant Pathol. 2020 Jul 28;21(9):1248–54. doi: 10.1111/mpp.12975 (PMC7411660; doi:10.1111/mpp.12975)
Supplement: Supplementary file 2 — FIGURE S2 [file MPP-21-1248-s002.pdf]

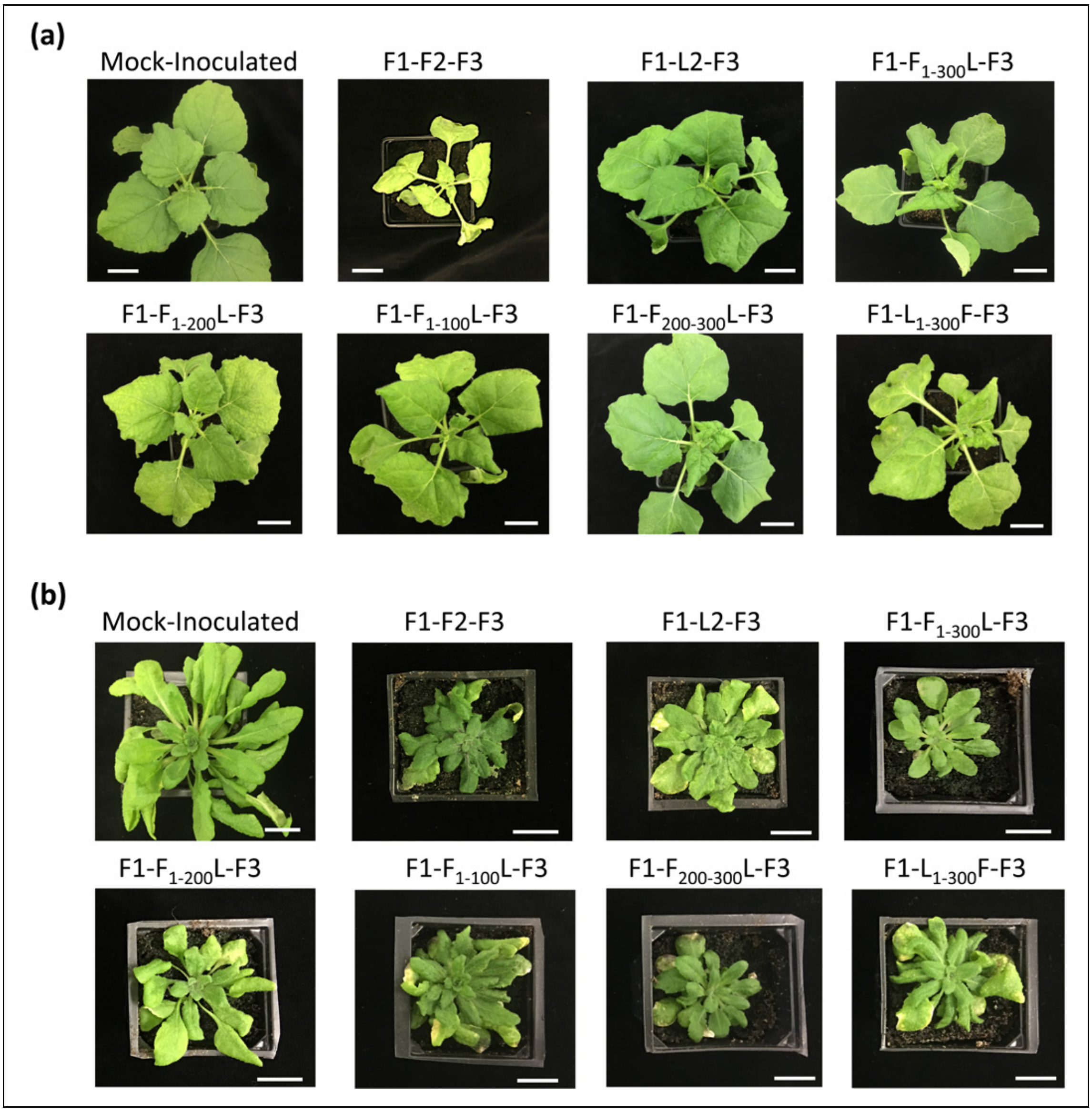

Fig. S2 Systemic disease symptoms on plants infected with reconstituted viruses and viral reassortant, and recombinant viruses. The Fny strain of cucumber mosaic virus (Fny-CMV) reconstituted by mixing synthetic RNAs generated by *in vitro* transcription of clones for Fny-CMV RNAs 1, 2, and 3 (F1-F2-F3) induced stunting, leaf deformation, and chlorosis in *Nicotiana benthamiana* plants (a) and stunting and leaf deformation in plants of *Arabidopsis thaliana* Col-0 (b). A reassortant virus constituted of the RNAs 1 and 3 of Fny-CMV and LS-CMV RNA2 (F1-L2-F3) induced milder disease symptoms in both host plants, as reassortant viruses possessing recombinant RNAs 2 possessing sequences derived from the RNAs 2 of LS-CMV and Fny-CMV (described in Fig. 1). *N. benthamiana* plants were inoculated with synthetic viral RNA mixtures on lower leaves 2 weeks after germination, and plants photographed 11 days later (a). *A. thaliana* plants were inoculated with purified virions (800 ng.μl^{-1}) at the 2-3 leaf stage, and photographed 22 days later. Mock-inoculated plants were mechanically inoculated with sterile water. Scale bars represent 2cm.
