# Supplementary material for: Effects of the cucumber mosaic virus 2a protein on aphid–plant interactions in Arabidopsis thaliana
Source: Mol Plant Pathol. 2020 Jul 28;21(9):1248–54. doi: 10.1111/mpp.12975 (PMC7411660; doi:10.1111/mpp.12975)
Supplement: Supplementary file 3 — FIGURE S3 [file MPP-21-1248-s003.pdf]

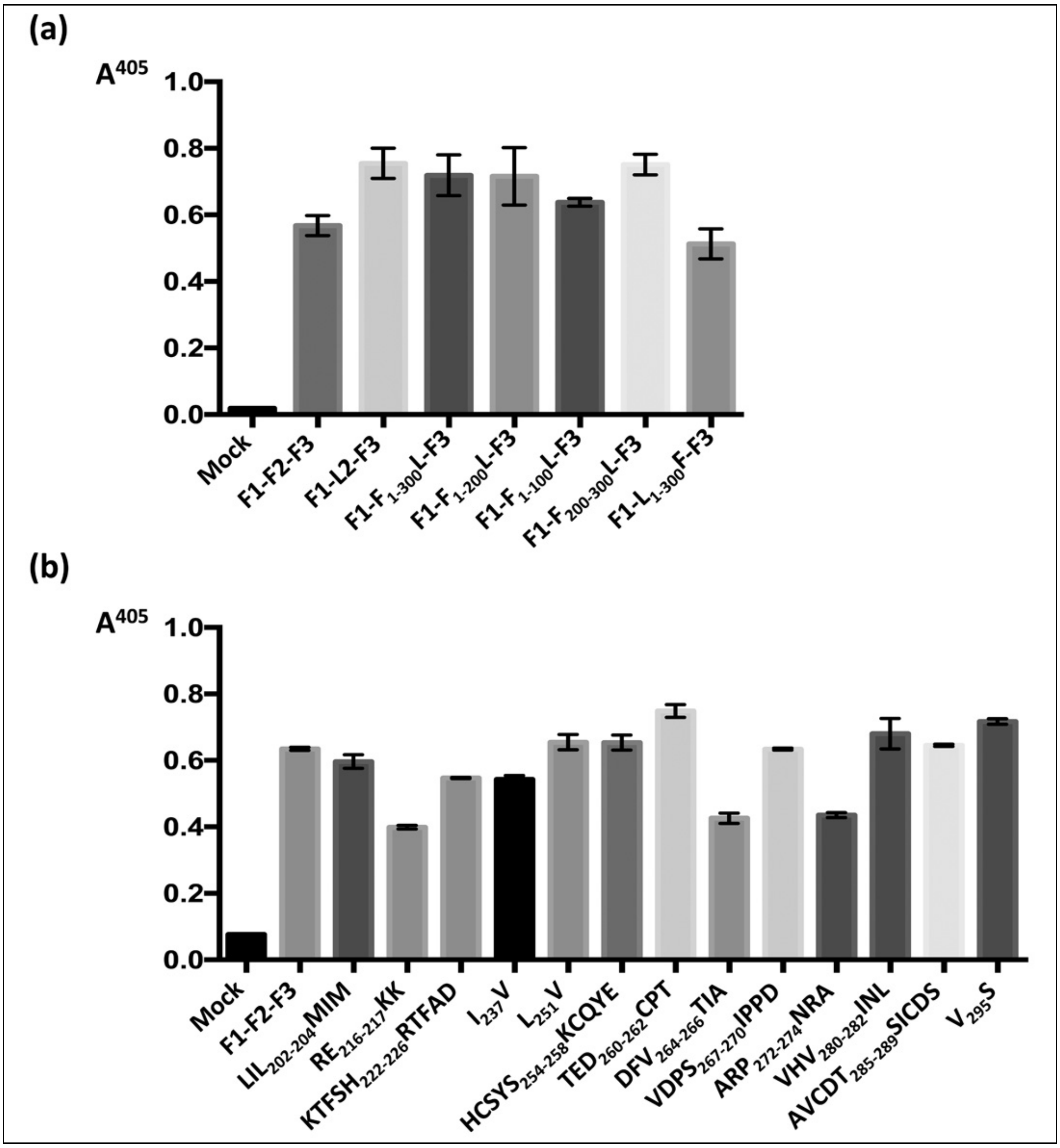

Fig. S3 Virus accumulation in plants of *Arabidopsis thaliana* Col-0 infected reassortant and recombinant cucumber mosaic virus derivatives. Double antibody sandwich enzyme-linked immunosorbent assays (DAS-ELISA) was used to determine relative levels of accumulation of cucumber mosaic virus (CMV) and inter-strain reassortant and recombinant viruses. Fny-CMV was reconstituted by mixing synthetic RNAs generated by *in vitro* transcription of clones for Fny-CMV RNAs 1, 2, and 3 (F1-F2-F3), and a reassortant virus comprising the RNAs 1 and 3 of Fny-CMV and LS-CMV RNA2 (F1-L2-F3) made by mixing appropriate synthetic viral RNAs. Panel (a) compares accumulation of F1-F2-F3 and F1-L2-F3 with reassortant viruses possessing recombinant RNAs 2 possessing sequences derived from the RNAs 2 of LS-CMV and Fny-CMV (described in Fig. 1). Panel (b) compares accumulation of F1-F2-F3 and F1-L2-F3 with F1-L2-F3 variants made using LS-CMV RNAs 2 with site-directed mutations in the 2a open reading frame. These mutants are described in Fig. 1c and Table 1. Optical absorbance at 405nm (A^{405}) indicates secondary antibody-linked horseradish peroxidase activity and relative accumulation of CMV coat protein. Each bar represents the mean using three plants per treatment, with standard error around the mean indicated by bars. No significant differences in virus accumulation were observed for any of the viral constructs.
