# Supplementary material for: Effects of the cucumber mosaic virus 2a protein on aphid–plant interactions in Arabidopsis thaliana
Source: Mol Plant Pathol. 2020 Jul 28;21(9):1248–54. doi: 10.1111/mpp.12975 (PMC7411660; doi:10.1111/mpp.12975)
Supplement: Supplementary file 4 — FIGURE S4 [file MPP-21-1248-s004.pdf]

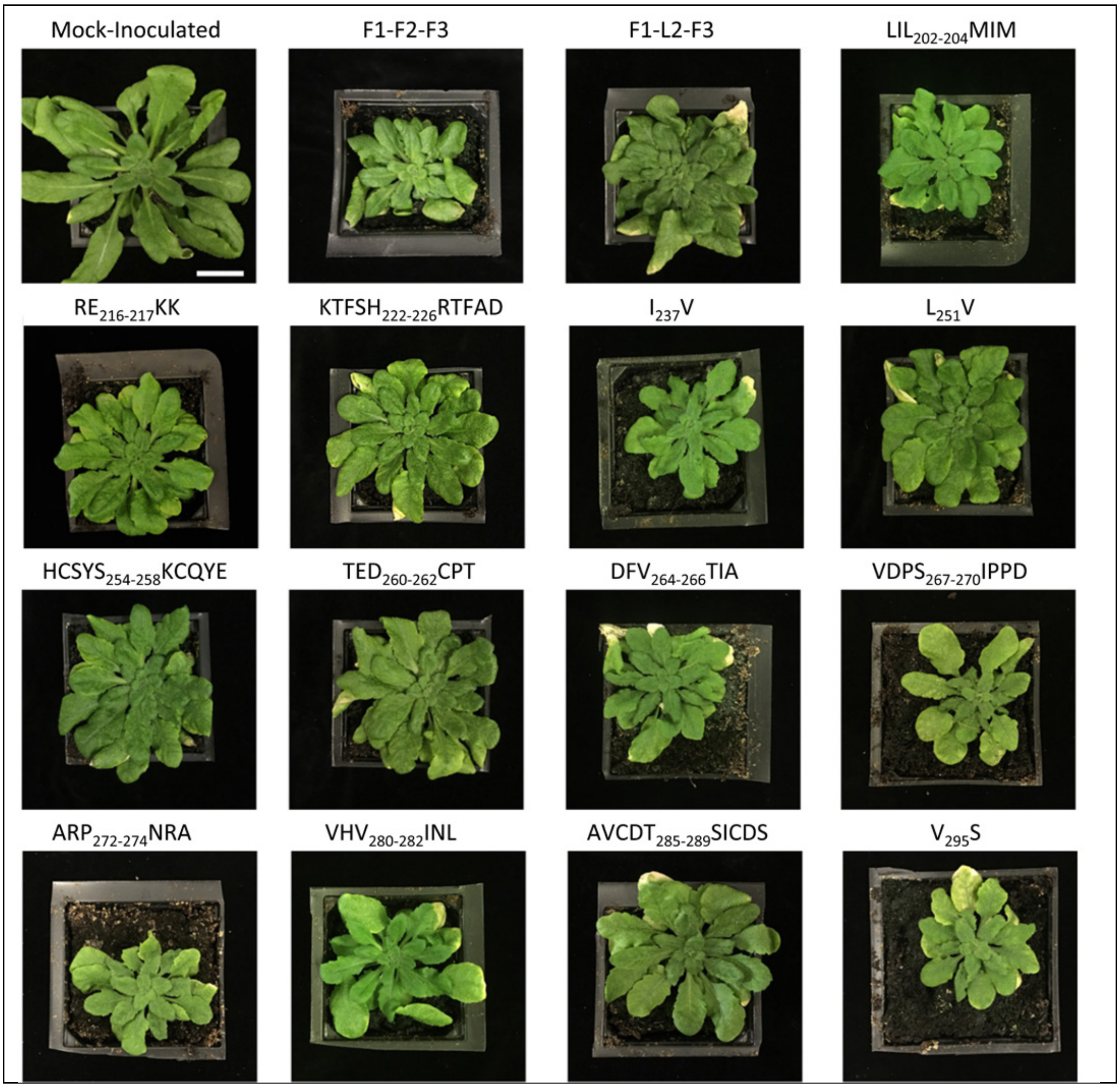

Fig. S4 Systemic disease symptoms induced by site-specific RNA2 mutants on plants of *Arabidopsis thaliana* Col-0. Plants were infected with the Fny strain of cucumber mosaic virus (Fny-CMV) reconstituted by mixing synthetic RNAs generated by *in vitro* transcription of clones for Fny-CMV RNAs 1, 2, and 3 (F1-F2-F3), a reassortant virus constituted of the RNAs 1 and 3 of Fny-CMV and LS-CMV RNA2 (F1-L2-F3), or F1-L2-F3 variants made using LS-CMV RNA2 site-directed mutants. In these mutants, the 2a ORF was altered so that specific amino acid sequences of the LS-CMV 2a protein lying between residues 201 and 300 were exchanged for corresponding residues of the Fny-CMV 2a protein. These mutants are described in Fig. 1c and Table 1. Among these mutants there was some variation in the degree of stunting and leaf deformation form that induced by F1-L2-F3, suggesting that amino acids between residues 201 and 300 of the CMV 2a protein might influence symptom development. Plants were inoculated with purified virions (800 ng.μl^{-1}) at the 2-3 leaf stage, and photographed 22 days later. Scale bars represent 2cm.
