# Supplementary material for: Effects of the cucumber mosaic virus 2a protein on aphid–plant interactions in Arabidopsis thaliana
Source: Mol Plant Pathol. 2020 Jul 28;21(9):1248–54. doi: 10.1111/mpp.12975 (PMC7411660; doi:10.1111/mpp.12975)
Supplement: Supplementary file 5 — TABLE S1 [file MPP-21-1248-s005.docx]

**Table S1.** Primer sequences for construction and validation of cucumber mosaic virus (CMV) RNA 2 clones and chimeric Fny/LS RNA 2 clones.

| **Objective** | **Primer name** | **Primer DNA sequences (5’ - 3’)** | **Processes** |
| --- | --- | --- | --- |
| Re-synthesis of Fny-CMV RNA 2 clone | SJ 9 | ACAATTTCACACAGGAAACAGCTAT | Synthesis of 5’-nontranslated region (NTR) of Fny-RNA 2 |
|  | SJ 10-1 | CGTCTTTGTAGTCCAT AGAAAAGATACTAGAAAGAAAAGATT |  |
|  | SJ 11 | ATGGACTACAAAGACGATGACGACAAG GCTTTCCCTGCCCC | Introduction of FLAG TAG at N-terminus of Fny 2a* |
|  | SJ 12 | GATATCAACACGATCAGTAGCACGG |  |
|  | SJ 13 | ATTA GGTACC TAATACGACTCACTATAG | Nested PCR |
|  | SJ 14 | AAATCCGGAGGAATCGCGATG |  |
| Re-synthesis of LS-CMV RNA 2 clone | SJ 15 | CACCCCAGGCTTTACACTTTATGC | Synthesis of 5’-nontranslated region (NTR) of LS-RNA 2 |
|  | SJ 16-1 | CGTCTTTGTAGTCCAT AGTAACAGAAAAGAGACTAG |  |
|  | SJ 17 | ATGGACTACAAAGACGATGACGACA ACAAGTCCTCCACCC | Introduction of FLAG TAG at N-terminus of LS 2a* |
|  | SJ 18 | ACGCGTCAACGACTGGTGTAAC |  |
|  | SJ 19 | TGTGGAATTGTGAGCGGATAACAA | Nested PCR |
|  | SJ 20 | GAGCTGTAGTGACGGTTTGCG |  |
| Synthesis of LS-/Fny-CMV chimeric RNA 2 clone F_1-300_L | SJ 25 | ATGGACTACAAAGACGATGACGACAAG | Introduction of 900bp of Fny 2a ORF sequence |
|  | SJ 27 | ACAAAGACTTGATGAAA AGAGTCGTCGTAATTACT |  |
|  | SJ 30 | TTTCATCAAGTCTTTGTGGAC | Amplification of LS 2a ORF |
|  | SJ 50 | GAGA TTAATTAA TCAGGAAACCAATCCAC |  |
|  | SJ 19 | TGTGGAATTGTGAGCGGATAACAA | Nested PCR |
|  | SJ 40 | GACTTTTGTAACCCCTCGCTCG |  |
| Synthesis of LS-/Fny-CMV chimeric RNA 2 clone F_1-200_L | SJ 28 | ATACAAAATCAAGTC GCACGCTTCACAAATG | Introduction of 600bp of Fny 2a ORF sequence |
|  | SJ 31 | GACTTGATTTTGTATCAATGT | Amplification of LS 2a ORF sequence |
| Synthesis of LS-/Fny-CMV chimeric RNA 2 clone F_1-100_L | SJ 29 | CCGATACGCAAGGGTTTGACCAT CGTCTCAAAAGCGAC | Introduction of 300bp of Fny 2a ORF sequence |
|  | SJ 32 | ATGGTCAAACCCTTGCGTATC | Introduction of LS 2a ORF sequence |
| Synthesis of LS-/Fny-CMV chimeric RNA 2 clone F_200-300_L | SJ 35 | GTACATGATCATGTC ACATTTAGCACAAATAGG | Introduction of 600bp of LS 2a |
|  | SJ 34 | ATTTGTGCTAAATGT GACATGATCATGTACCAG | Introduction of 600bp-900bp of Fny 2a |
|  | SJ 27 | ACAAAGACTTGATGAAA AGAGTCGTCGTAATTACT |  |
| Synthesis of LS-/Fny-CMV chimeric RNA 2 clone L_1-300_F | SJ 54 | AGGGACATCAGCATTTCTTTT | LS 2a |
|  | SJ 36 | GAACACTTGATGAAA GGAATCATCGTAGTTAA | Introduction of 900bp of LS 2a ORF sequence |
|  | SJ 37 | TTTCATCAAGTGTTCGTCGAAAGTGC | Introduction of Fny 2a ORF sequence |
|  | SJ 38-1 | CATGTGGTCATAAAACTGCAAATTCTC |  |
|  | SJ 39-1 | CCGGAAGATCATCATAAGAAAGACCTG | Nested PCR |

* To facilitate future work on 2a protein function we fused in-frame sequences encoding FLAG tags to the 5’ region of the 2a ORFs for Fny-CMV and LS-CMV encoded by the plasmids pFny206 and pLS-CMV2 (Rizzo and Palukaitis, 1990 *Mol. Gen. Genet.* **222,** 249-256; Zhang *et al*., 1994 *J. Gen. Virol.* **75**, 3185-3191).
