# Supplementary material for: Effects of the cucumber mosaic virus 2a protein on aphid–plant interactions in Arabidopsis thaliana
Source: Mol Plant Pathol. 2020 Jul 28;21(9):1248–54. doi: 10.1111/mpp.12975 (PMC7411660; doi:10.1111/mpp.12975)
Supplement: Supplementary file 6 — TABLE S2 [file MPP-21-1248-s006.pdf]

Table S2. Sequences of mutagenic primers used for site-specific mutagenesis of the cucumber mosaic virus 2a open reading frame.

Primer	Mutagenic Primer DNA sequence (5' - 3')	Amino acid substitutions in 2a ORF/<br>Nucleotide substitutions in viral RNA sequence
SDM1-F	ATCATGTATCAATGTCCGTGTTTC	KTFSH → RTFAD<br>AGGACCUUCGCUGAU → AAAACAUUCUCUCA
SDM1-R	CATGTCACATTTAGCACACAAATAGGG	
SDM2-F	GGTCTTTTCATATTGACACTTGACAGGTAACAA	KCQYE → HCSYS<br>CAUUGUUCUUAUUCA → AAGUGUCAAUAUGAA
SDM2-R	CCTGTCAAGTGTCAATATGAAAAGACCGAGGA	
SDM3-F	GCGATTTCGTCATTCCTCCGGATCTTGCGC	VDPS → IPPD<br>GUUGAUCCUAGU → AUUCCUCCGGAU
SDM3-R	CCTCCGGATCTTGCGCGACCTACTG	
SDM4-F	TATCAGTAGCACGGTTTAAACTAGGATCAACG	ARP → NRA<br>UUGCGCGACCUA → UUAAACCGUGCU
SDM4-R	CCTAGTTTAAACCGTGCTACTGATAGGGTTG	
SDM5-F	GCAGAGTCGAGTCACAAATGGATTGAACTACATGTA	AVCDT → SICDS<br>GCCGUUUGCGAUACC → UCCAUUUGUGACUCG
SDM5-R	AGTTCAATCCATTTGTGACTCGACTCTGCCCACCCATG	
SDM6-F	ATCATGTATCAATGTCCGTGTTTC	LIL →MIM<br>GACUUGAUUUUG → GACAUGAUCAUG
SDM6-R	CATGTCACATTTAGCACAAATAGGG	
SDM7-F	TTTAGTTCAAGCCGTTTGCGAT	VHV → INL<br>GUACAUGUA → AUCAAUUUA
SDM7-R	TTGATATCAACCCTATCAGTAGGTC	
SDM8-F	ACCATTTTTGGTACCTGTCCATTG	L → V<br>UUA → GUA
SDM8-R	CCCAAATTTGATAACAGCG	
SDM9-F	CGCAAGATTTCTTAAGTGCGTTAAAATC	RE→ KK<br>CGUGAG → AAGAAA
SDM9-R	CGCACTTAAGAAATCTTGCGCAGAG	
SDM10-F	GCCCACCCATAGTAACTACGATG	V → S<br>GUU → AGU
SDM10-R	AGAGTGGTATCGCAAACG	
SDM11-F	CGATGGTGTCGTTGATAACGC	I →V<br>AUU → GUU
SDM11-R	AGACCCTCGATAACATAG	
SDM12-F	AGGACCCCACCATCGCGGTTGATCCTAGTC	DFV → TIA<br>GAUUUCGUC → ACCAUCGCG
SDM12-R	GGATCAACCGCGATGGTGGGGTCCTCGGTC	
SDM13-F	TTCAAAGTGTCCAACGCCCGATTTCGTCGTTG	TED → CPT<br>ACCGAGGAC → UGUCCAACG
SDM13-R	AAATCGGGCGTTGGACACTTTGAATAAGA	
