# Supplementary material for: Effects of the cucumber mosaic virus 2a protein on aphid–plant interactions in Arabidopsis thaliana
Source: Mol Plant Pathol. 2020 Jul 28;21(9):1248–54. doi: 10.1111/mpp.12975 (PMC7411660; doi:10.1111/mpp.12975)
Supplement: Supplementary file 7 — METHOD S1 [file MPP-21-1248-s007.docx]

**SUPPORTING INFORMATION**

**Title: Effects of the cucumber mosaic virus 2a protein on aphid-plant interactions in *Arabidopsis thaliana***

**Authors: Sun-Ju Rhee, Lewis G. Watt, Ana Cazar Bravo, Alex M. Murphy, and John P. Carr**

Department of Plant Sciences, University of Cambridge, Downing Street, Cambridge CB2 3EA, United Kingdom

**Supplementary Methods**

**Plants, aphids and aphid performance assays**

Plants of *Nicotiana benthamiana* Domin., Chinese cabbage (*Brassica rapa* subsp. *pekinensis* L*.*), and *Arabidopsis thaliana* Heyn. accession Col-0 were grown under previously described conditions (Lee *et al*., 2016; Westwood *et al*., 2013). Transgenic plants used in this study have been described by Westwood and colleagues (2013). Wingless *Myzus persicae* Sulzer clone US1L (Devonshire & Sawicki, 1979) were maintained on Chinese cabbage. For aphid performance experiments, adults were placed onto leaves of healthy Chinese cabbage plants and allowed to reproduce for no longer than 24 hours. Nymphs produced during this time were transferred to Arabidopsis plants using fine paintbrushes and confined on these plants using micro-perforated plastic bags (Associated Packaging, Kent, UK) (Tungadi *et al*., 2020; Westwood *et al.,* 2013).

Two types of performance assays were used: measurement of mean relative growth rate (MRGR) of aphid nymphs, and measurement of aphid colony growth. For determination of MRGR, one-day-old aphid nymphs were weighed on an MX5 microbalance (Mettler-Toledo Inc., Columbus, OH, USA) and placed onto mock-inoculated or virus-infected Arabidopsis plants (Westwood *et al*., 2013). Arabidopsis plants were used at 10 days following viral inoculation or mock inoculation. Six days later each aphid was re-weighed again and MRGR calculated using the formula: MRGR = (log_e_W_final_ - log_e_W_initial_)/t, where t = time (days) between measurement of aphid fresh weight (W) (Stewart *et al.*, 2009, Leather and Dixon, 1984). To measure colony growth (reproductive performance) nymphs were placed singly onto infected or mock-inoculated Arabidopsis plants and the offspring numbers counted 10 days later. At least 35 technical replicates per treatment group were performed and each experiment was carried out at least three times (biological replicates). Analysis of variance (ANOVA) followed by Dunnett’s *post hoc* multiple comparisons (n ≥ 35, ****p < 0.0001, 0.0001 <***p < 0.001, 0.001<**p< 0.01<, *p<0.01) used R (Dalgaard, 2008).

**Construction of chimeric infectious RNA 2 clones**

Using the plasmids pFny206 and (Rizzo and Palukaitis, 1990) and pLS-CMV2 (Zhang *et al.*, 1994), as backbones five recombinant RNA 2 infectious clones encoding chimeric 2a proteins were constructed as follows: F_1-300_L (encodes a chimeric 2a protein with residues 1-300 derived from the Fny-CMV 2a protein); F_200_L (encodes a chimeric 2a protein with residues 1-200 derived from the Fny-CMV 2a protein); F_100_L (encodes a chimeric 2a protein with residues 1-100 derived from the Fny-CMV 2a protein); F_200-300_L (encodes a chimeric 2a protein with residues 200-300 of the Fny-CMV 2a protein inserted into the LS-CMV 2 backbone), and L_1-300_F constructed by insertion of the sequence encoding residues 1-300 of the LS-CMV 2a protein into Fny-CMV RNA 2 clone (Fig. 1b).

Clones encoding Chimeric clones were generated in three steps using PCR with Phusion HF DNA polymerase (Thermo Fisher Scientific) with appropriate primers (Table S1). DNA encoding the LS-CMV RNA 2 5’-NTR was amplified using primers SJ 15 and SJ 16-1 (in case of L_1-300_F: Fny-5’NTR/primer: SJ 9 and SJ 10-1), Fny-CMV 2a coding sequence by SJ 25 and SJ 40 (LS 2a/ primer: SJ 25 and SJ 36), and the rest of the LS-CMV 2a coding sequence to construct F_1-300_L was amplified using SJ 30 and SJ 50 (the rest of the Fny 2a/ Primer: SJ 37 and SJ 38-1) (Table S1).

In the second step, we performed overlap PCR using primer SJ 19 and SJ 27 (SJ and SJ 36) with the PCR products of LS-5’NTR (Fny-5’NTR) and the Fny 2a (the LS 2a) as templates. The resulting fragment was used for a final overlap PCR using primers SJ 13 and SJ 27 (SJ 13 and SJ 39-1) with the PCR products of rest of LS 2a which amplified in the first step (the rest of Fny 2a) as templates. The final overlap PCR products and LS-CMV RNA 2 backbone were digested with *Bam*HI and *Sal*I and ligated. To generate L_1-300_F, *Kpn*I and *Xba*I were used for sub-cloning into the plasmid encoding Fny-CMV RNA 2.

The plasmids F_1-200_L, and F_1-100_L were generated in two steps using PCR. One of the fragments for cloning F_1-200_L and F_1-100_L was amplified from the 600bp (SJ 25 and SJ 28) and 300bp (SJ 25 and SJ 29) of the Fny 2a, respectively. The other fragments were amplified of LS 2a region positioned from next to the end of the amplified-Fny 2a using SJ 31/SJ 50 (for F_1-200_L) and SJ 32/SJ 50 (for F_1-100_L). Subsequently using PCR products of corresponding Fny 2a and LS 2a were performed overlap PCR by SJ 25 and SJ 40. They are digested with *Sal*I and ligated into the *Sal*I-digested F_1-300_L plasmid. To construct F_200-300_L, PCR amplified from 5’NTR of to 600bp of the LS-CMV RNA 2 region using SJ 15 and SJ 35. The other fragment was amplified from 601bp of LS 2a using SJ 34 and SJ 50. Overlap PCR was performed using SJ 25 and SJ 40. The resulting DNA molecule was ligated into *SnaB*I/*Sal*I-digested plasmid encoding LS-CMV RNA 2. Site-specific mutants were made using the Q5 site-directed mutagenesis kit (New England Biolabs). Mutagenic primer information is presented in Table S2. All chimeric DNA clones were authenticated by automated Sanger sequencing (Source Bioscience Ltd., Cambridge, UK). To facilitate future work on 2a protein we fused in-frame sequences encoding N-terminal FLAG tags into the Fny-CMV and LS-CMV 2a coding sequences.

**Preparation of viral inoculum**

Infectious RNA 2 molecules were generated by *in vitro* transcription of 1μg of linearized plasmid DNA using T7 RNA polymerase using the mMESSAGE mMACHINE T7 kit (Ambion). Plasmids encoding Fny-CMV RNA 1, Fny-CMV RNA 2, L_1-300_F, and Fny-CMV RNA 3 were linearized using *Pst*I. Plasmids encoding LS-CMV RNA 2, F_1-300_L, F_1-200_L, F_1-100_L, and F_200-300_L were linearized using *Hind*III. Transcripts of Fny-RNA 1, chimeric RNAs 2, and Fny-RNA 3 mixed (1:1:1) and mechanically inoculated onto lower leaves of 2 week-old *N. benthamiana* plants using Carborundum as an abrasive. Passaging of virus in *N. benthamiana* leaves was done by mechanical inoculation using extracts of systemically-infected leaves homogenized in 0.01M phosphate buffer pH7.4. Virions were purified according to Palukaitis (2019) using 10 - 30g of systemically infected *N. benthamiana* leaves. Purified virions (800 ng/μl) were inoculated onto Carborundum-dusted Arabidopsis Col-0 leaves for the aphid experiment.

**Detection and analysis of viruses in plant tissues**

Virus titers were estimated using double sandwich enzyme linked immunosorbent assay kits for CMV (Bioreba AG). Arabidopsis leaf tissue samples 100mg from three individual plants were pooled for each assay and the experiment replicated least three times. To authenticate that mutant sequences were stable in planta total RNA isolated from non-inoculated leaves of virus-infected *N. benthamiana* and Arabidopsis plants using the RNeasy Plant Mini Kit (Qiagen). Using 2μg of total RNA, first-strand cDNA was synthesized using Superscript RTaseIII (Invitrogen) with random hexamer. Primer sets for RT-PCR and sequencing were designed close to the junction of the Fny-CMV 2a coding sequence with the LS-CMV 2a coding sequence. RT-PCR was carried out on 1μl of cDNA using Phusion DNA polymerase (Thermo Fisher Scientific), and primers SJ 25/SJ 54 or SJ 25/SJ 38-1 (Table S1). RT-PCR conditions are as follows; 98 °C for 30 s, 35 cycles of 98 °C for 10 s, 60°C for 30 s, and 72 °C for 1 min, with a final extension at 72 °C for 2 min. PCR amplicons were subjected to automated Sanger sequencing.

**References**

**Dalgaard, P.** (2008) Descriptive statistics and graphics. In: *Introductory Statistics with R.* *USA: Springer*, 67-94.

**Devonshire, A.L. and Sawicki, R.M.** (1979) Insecticide-resistant *Myzus persicae* as an example of evolution by gene duplication. *Nature,* **280,** 140-141.

**Leather, S. and Dixon, A.** (1984) Aphid growth and reproductive rates. *Entom. Exp. Appl.* **35,** 137-140.

**Palukaitis, P.** (2019) Virus purification and RNA isolation. In: Palukaitis, P., García-Arenal, F., eds. *Cucumber Mosaic Virus* USA: *American Phytopathological Society*, 219-225.

**Rizzo, T. M. and Palukaitis, P.** (1990) Construction of full-length cDNA clones of cucumber mosaic virus RNAs 1, 2 and 3: generation of infectious RNA transcripts. *Mol. Gen. Genet.* **222,** 249-256.

**Stewart, S. A., Hodge, S., Ismail, N., Mansfield, J. W., Feys, B. J., Prospéri, J.-M., Huguet, T., Ben, C., Gentzbittel, L. and Powell, G.** (2009) The *RAP1* gene confers effective, race-specific resistance to the pea aphid in *Medicago truncatula* independent of the hypersensitive reaction. *Mol. Plant-Microbe Interact.* **22,** 1645-1655.

**Westwood, J.H., Groen, S.C. Du, Z., Murphy, A.M., Anggoro, D.T., Tungadi, T., Luang-In, V., Lewsey, M.G., Rossiter, J.T., Powell, G. P., Smith, A.G. and Carr, J. P.** (2013) A trio of viral proteins tunes aphid-plant interactions in *Arabidopsis thaliana. PLoS One,* **8**, e83066.

**Zhang, L., Handa, K. and Palukaitis, P.** (1994) Mapping local and systemic symptom determinants of cucumber mosaic cucumovirus in tobacco. *J. Gen. Virol.* **75,** 3185-3191.
